# Fit to the rasch model of the FEEL-KJ Spanish adaptation

**DOI:** 10.1016/j.heliyon.2023.e21435

**Published:** 2023-10-30

**Authors:** Rodrigo M. Pazos Siri, Catalina P. Morales-Murillo, Mª Dolores Grau Sevilla, Adoración-Reyes Moliner

**Affiliations:** a Escuela de Doctorado, Catholic University of Valencia “San Vicente Mártir”, Valencia, Spain; b Faculty of Psychology, Catholic University of Valencia “San Vicente Mártir”, Valencia, Spain; c Equipo de Investigación en Atención Temprana-INAT, Universidad Internacional de La Rioja, Logroño, Spain

## Abstract

**Background:**

In recent decades, scientific production related to Emotional Regulation (ER) has increased significantly. Deficits in ER have been linked to various mental health problems. The aim of this work is to evaluate the Spanish adaptation of the Feel-Kj scale to the RASCH model.

**Method:**

Children from 9 to 16 years old took part of this study. One hundred and eighteen children between 9 and 16 years old and one hundred and fifteen from 13 to 16. The 254 participants were attending 25 schools located in de Valencian Community in Spain.

**Results:**

Both infit and outfit MNSQ statistics provided evidence for the construct validity of the FEEL-KJ questionnaire. The infit and outfit mean values (1.01 and 1.02, respectively) were close to the perfect fit value of 1.

**Conclusions:**

From these results it can be sensed that the FEEL-KJ will be a valid and reliable instrument to apply in the Spanish speaking population, although it is necessary to make some minor adjustments in terms of translation.

## Teaser

What this paper adds? This article provides a new tool in Spanish language to obtain information about which strategies children and adolescents use to regulate anxiety, fear, and anger. It also classifies these strategies into adaptive, maladaptive, and external. This information can be very relevant to identify patterns of emotion regulation characterized by the use of maladaptive strategies that may be associated with various psychological pathologies.

## 1 Introduction

In recent decades, scientific production related to Emotional Regulation (ER) has increased significantly [1]. ER is the process through which we influence emotions; not only in terms of which ones we experience, but also when we experience them and how we experience and express them [2]. Deficits in ER have been linked to various mental health problems, such as Border Personality Disorder, Depression, Generalized Anxiety Disorder, Substance Abuse, Food Behaviour Disorders and Somatoform Disorders [[3], [4], [5], [6]].

The body of knowledge related to Emotional Regulation not only links this construct with various psychopathologies, but also found an important influence at the level of social functioning [7].

At the childhood level psychopathological symptoms in children are also related to a lower use of adaptive emotional regulation strategies [8]. Adaptive emotional regulation strategies relate to academic success, better social functioning and psychological and physical wellbeing, both in adolescence and infancy [9].

While there seems to be some consensus on the relevance of the ER study, research in this area proposes several challenges. The concept of ER itself is complex, starting from the very notion of what an emotion is versus what the process of emotional regulation is [1]. Berking and Wpperman (2012) point to the difficulties presented using ER as an experimental concept, due to the need to study the different ER strategies depending on the different emotions that are being regulated. These authors propose that research be oriented to establish the relationship between regulatory strategies and concrete emotions, thus avoiding taking the ER as if it were a unique process independent of the emotions that are its object. In this regard, Theurel & Gentaz (2018) found that using reappraisal seems to be more suitable than distraction for reducing fear or anxiety. Their results also indicate that using reappraisal is not so effective for reducing sadness or self-conscious emotions [10]. [11] found that there is strong evidence for a significant relation between threat reappraisal and anxiety symptoms severity reduction. It was also found that recalling an event from a third-person perspective facilitated anger control whereas recalling an event from own perspective or using no strategy had less of an impact in regulating anger [12]. [13] Found that strategies used both by male and females differ when they attempt to regulate anger or sadness.

These findings support the challenges identified by Berking and Wpperman (2012), because they indicate that different ER strategies vary their results when applied to different emotions. To respond to these challenges, it seems important to have ER assessment tools that address these issues.

As a result of the increase in interest in the study of the ER, a wide variety of tools have been generated for its assessment [14]. Below are three of the most cited tools adapted to Spanish for the study of the ER.

The CERQ (Cognitive Emotion Regulation Questionnaire) (Garnefski and Kraij [15], was adapted to Spanish by Domínguez-Sánchez et al. [16]. This tool measures the cognitive emotional regulation strategies used when faced with stressful situations. Blaming others, catastrophism, putting oneself in perspective, positive re-evaluation, planning, positive reorientation, rumination, acceptance and self-blame are the nine sub-scales that correspond to nine strategies of emotional regulation (Garnefski & Kraaij, 2007): self-blame, refers to thoughts that tend to place responsibility for the experience in the person himself; acceptance is referred to the presence of thoughts of acceptance and resignation to the event experienced, rumination refers to thinking about the thoughts or actions that have been associated with the event; positive reorientation refers to thinking about happy and nice situations rather than focusing about the event being experienced, planning refers to thinking about the steps to follow and how to manage the negative event, positive re-evaluation refers to thoughts oriented to a positive meaning of the event in terms of personal growth, putting oneself in perspective refers to thoughts that detract from importance or emphasize relativity when compared to the event with other events, catastrophism refers to thoughts that explicitly emphasize the terror of what has been experienced, and blaming others refers to thoughts that place responsibility for what is experienced in the environment or in another person. Putting into perspective, positive reappraisal, refocus on planning, positive refocusing and acceptance sub-scales are grouped into de adaptive strategies category, while catastrophizing, other blame and self-blame sub-scales are grouped in the unadaptive strategies category. Each of the sub-scales consists of a questionnaire of 4 items on the Likert scale 1st "almost never" to 5th "almost always"). The score obtained in the tool indicates the frequency of the use of the strategy, the higher the score, the more frequent the use of the strategy. The psychometric properties in both the English and Castilian version are satisfactory. The Castilian version gets a Cronbach's Alpha from 0.61 to 0.89. CERQ is a useful instrument in that it envisages a wide range of regulatory strategies, but merely assesses the use of such strategies in stressful situations, without reference to which strategies are used to regulate specific emotions. So, in this regard it does not help to answer to challenges regarding how certain ER strategies relate to emotions.

The Difficulties in Emotion Regulation Scale (DERS) [17] is another widely used instrument that also has been adapted to the Spanish language [18]. DERS is a self-reporting questionnaire that measures difficulties in clinically relevant emotion regulation. The Adaptation to Spanish of this scale consists of the subscales emotional control, daily interference, emotional neglect, emotional rejection, and emotional confusion. This adaptation has a good level of internal consistency (alpha between .73 and .71) and good test-retest reliability in a 6-month period (p.74, p<,001). The scale evaluates variables that interfere with successful emotional regulation, providing highly relevant information at the clinical level. However, its goal is not to provide information about which regulation strategies are used, or how using these strategies is related to the ER process.

The Emotion Regulation Questionnaire (ERQ) is a questionnaire elaborated by Ref. [19] that evaluates the use of cognitive re-evaluation and suppression strategies. The adaptation to Spanish was made by Ref. [20]. The ERQ consists of 10 items, grouped into a factorial structure of 2 factors, 6 corresponding to the cognitive re-evaluation factor and 4 to the suppression factor. This questionnaire’s Spanish adaptation has shown good psychometric properties (alpha .75; .82) and (alpha .68, .76) [21]. This instrument values the use of two regulatory strategies that its authors identify as the most used, but does not value the use of such strategies based on the emotion that the person is trying to regulate and it does not take into consideration other ER strategies that could be relevant.

The Feel-KJ [22] would be a great complement to the existing toolkit, as it measures fifteen strategies in relation to different emotions (anxiety, sadness and anger) and also classifies them into adaptive, unadaptive and external regulation strategies. The information provided by this instrument is interesting because it responds to the limitation mentioned in Berking and Wpperman's article, which refer to the need to study the use of emotion regulation strategies in relation to the emotional states that need regulation (Berking & Wupperman, 2012).

In the Spanish language, within the knowledge of the authors of this work, there is no instrument to provide this information. The validation of the Feel-KJ has been carried out in the German population, with a sample of 1446 children [22] and in a Belgian population of 1118 children between the age of 8 and 18 [23]. The factorial analysis of the Feel-KJ confirmed a two-factor structure (Grob & Smolenski, 2005); that were identified as adaptive strategies and maladaptive strategies. Adaptive strategies include Problem-Oriented Actions, Distraction, Mood Empowerment, Acceptance, Forgetfulness, Cognitive Problem Resolution, and Re-evaluation. Out-of-the-box strategies include Giving Up, Retiring, Rumination, Self-Devaluation, Aggression. It also includes an external regulation category that encompasses strategies such as expression, social support, and reverse emotional control.

This work focuses on achieving three objectives. First, to carry out a study that evaluates Feel-KJ’s item adjustment to de Rasch model, in order to assess the instrument’s suitability for the Spanish-speaking population. Secondly, it is sought to promote the dissemination of a methodology that is not frequently used in Spanish literature for this type of tools and thus promote an alternative for psychometric test validation that allows to overcome the limitations of classical test theory. The third objective is to expand the statistical support that the Feel-KJ has, providing information about the test′s adjustment to the theory of response to the items in the Spanish-speaking population.

Reasons to choose rash model over the classical test theory are two-fold, firstly because of its novelty in Spanish literature and second because its method of calculation is robust for small sample sizes.

## 2 Method

### 2.1 Participants

A convenience sample method was used to gather participants for this study. Once the project was approved by an Institutional Review Board, relevant school authorities were contacted to invite them to participate in the study. Once the school’s directors gave their consent, families were contacted by the school to take part in a meeting, where they were informed of the characteristics of the study. Parents who wished to take part, signed an informed consent. Permission was obtained to administrate the FEEL-KJ questionnaires during school hours and in the student's classrooms. Students were informed that participation in the study was voluntary and that no measures would be taken if they decided not to participate. All students older than 12 years old signed assent for their participation in the study, besides their parents signed an informed consent.

Children from 9 to 16 years old took part of this study. One hundred and eighteen children between 9 and 16 years old and one hundred and fifteen from 13 to 16 years old. Participants were recruited from 25 semi-private schools located in de Valencian Community in Spain. These Semi-private schools (called “centros concertados” in Spain) are run and paid by de state, but part of the running costs are provided by an affordable monthly parental contribution.

One-hundred and nine boys participated in this study and one-hundred and two participants were girls. Participant’s sex information was missing in 43 cases. In total two-hundred and fifty-four children participated in the study.

To participate in this study candidates had to be 10 years of age during the school year at the least, and turn16 years of age during the school year at the most. They also had to provide parental authorization by handing in the informed consent form signed by their parents/tutors. Children with an stablish diagnosis of intellectual disability were excluded from this study.

No participants where excluded due to errors or omissions in their responses. No child or family participated in this study without a signed consent. No outliers were identified in the dataset. Participants with missing data where handled leased wised. No counterbalancing technique was applied.

### 2.2 Instruments

The Feel-kj is a self-reporting instrument which comprises of 90 items through which the strategies used to regulate feelings of anxiety, sadness and anger are evaluated. Qualitative interviews with children were used to generate the questionnaire’s items. Interviewed children were between 4 and 16 years old. Each of the fifteen strategies is measured with 6 items, 2 items per strategy for the three emotions: anxiety, sadness, and anger. Each item comprises a Likert scale composed of five points, where the extreme values are “never” to “almost always”. Two major factors have been distinguished, which in turn are made up of several emotion regulation strategies.

The first factor, Adaptative Emotion Regulation is composed of the strategies shown in Table 1.

**Table 1:** Feel-KJ adaptive strategies and item numbers.

Strategy	Item number
**Problem Solving**	1
	18
**Distraction**	4
	27
**Humor Enhancement**	3
	17
**Acceptance**	6
	21
**Forgetting**	15
	20
**Cognitive Problem Solving**	11
	28
**Revaluation**	12
	29

The second factor, unadaptative strategies is composed of the strategies shown in Table 2.

**Table 2:** FEEL-KJ Unadaptive Strategies and item numbers.

Strategy	Item number
**Giving up**	9
	30
**Aggression**	13
	23
**Move away**	7
	25
**Self-evaluation**	8
	16
**Ruminate**	10
	16

The third factor, external regulation strategies is composed of items shown in Table 3.

**Table 3:** FEEL-KJ external regulation strategies and item numbers.

Strategy	Item number
**Social support**	2
	19
**Expression**	14
	22
**Emotional Control**	5
	26

Three of the strategies studied could not be classified as adaptive or unadaptive. In the instrument manual they are grouped under the category "External regulation strategies".

Regarding the secondary scale’s internal consistency, the unadaptive group of strategies had an internal consistency of α 0.82 and the group of adaptive strategies had an α of 0.93. Internal consistency for primary scales was good with a α 0.69 to 0.91. Test re-test confidence over 6 weeks is shown in the instrument’s manual and the tool also has an adequate correlation with symptoms of depression and anxiety. The instrument was translated (Dutch – Spanish) following the translation and retro transduction method [24,25] all items contained in the Dutch version were maintained and none had to be modified in the translation process.

Within the research project of which this article is a part, Sanchis-Sanchis et al. [26] carried out a correlation study between the scores their participants obtained in the Spanish version of the Feel-KJ and the Cognitive Emotion Regulation Questionnaire [15] Spanish version [16]. Results of this study showed positive and statistically significant correlations between the adaptive strategies factor scores of the FEEL-KJ and the adaptive dimension of the CERQ scores; and the maladaptive strategies factor scores of the Feel-KJ and the maladaptive dimension of the CERQ scores. These results would support the notion that FEEL-KJ’s Spanish translation measures emotional regulation. Therefore, supporting the Feel-KJ construct validity.

### 2.3 Procedure

This study is part of a larger one related to self-regulation in children and young people in the Valencian Community, Spain. Once the project was approved by an Institutional Review Board, relevant school authorities were contacted to make the invitation to families to participate. Parents who wished to take part, signed an informed consent. Permission was obtained to administrate the FEEL-KJ questionnaires during school hours and in the students' classrooms.

### 2.4 Data analysis

The data analysis software WINSTEPS [27] was used to run the different tests. To determine the reliability of the participant’s score obtained in the Feel-kj questionnaire, item and persons reliability and separation indexes were calculated. Indexes obtained for Reliability of items was >0.70 (KR-20 or Cronbach's Alpha) and a separation >2, indicate adequate internal consistency [28,29]. To assess the validity of the scale through item’s fit to the Rasch Model, the Infit and Outfit unstandardized information-weighted mean square statistics were calculated. Item fit values (i.e., infit and outfit) from 0.7 to 1.4 are considered a misfit [30,31]. Point-measure correlations (PMC) were used to assess if the items discriminate among different ability levels of participants self-regulation [32]. recommended PMC values between 0.3 and 0.7 for items to have good discrimination levels. Bond and Fox (2007) advised against taking items out of an instrument based on PMC values only. These authors explained that taking out any particular item could alter the PMC scores for other items, other statistics or parameters should also be considered.

The item level difficulty was determined by the measure logits. Item difficulty and persons' ability levels were compared through the person-item map [33]. Finally, the adequacy of the five-point category rating scale was tested through statistics (i.e., category frequency and monotonicity of average measure) and thresholds (i.e., step calibrations and category fit scores: Infit and Outfit MNSQ).

## 3 Results

The participants' FEEL-KJ scores were the highest for the emotion Anxiety (M x 88.11, SD s 13.77) and the lowest for Anger (M x 87.22, SD x 12.21). The scores for Adaptive Emotion Regulation ranged from 15.96 in revaluation to 20.92 in problem solving. As for Maladaptive Emotion Regulation, participants reported the lowest ratings in aggressive actions (12.17) and the highest for Rumination (18.22). Expression (14.79) was the strategy with the lowest and social support (18.61) was the highest in the subscale External Emotion Regulation. Table 4 summarizes the participants' mean scores in the FEEL-KJ questionnaire.

**Table 4:** Descriptive statistics by FEEL-KJ strategies, emotions, and dimensions.

Subscales and Dimensions	Min - Max	M (SD)
Emotions		
Anger	44–121	87.22 (12.21)
Sadness	46–121	87.92 (13.35)
Anxiety	20–131	88.11 (13.77)
Emotion Regulation Strategies		
Adaptive	40–207	136.09 (28.20)
Problem solving	6–30	20.92 (4.79)
Distraction	6–30	20.83 (6.32)
Forgetting	6–30	20.81 (6.20)
Acceptance	4–30	17.32 (4.61)
Humor Enhancement	4–30	19.69 (4.80)
Cognitive Problem Solving	6–30	20.57 (5.18)
Revaluation	4–30	15.96 (5.00)
Maladaptive	24–130	75.66 (16.22)
Giving Up	5–30	14.72 (4.73)
Withdrawal	4–30	13.24 (5.61)
Rumination	5–30	18.22 (4.82)
Self-Devaluation	6–30	17.31 (4.37)
Aggressive Actions	4–27	12.17 (4.72)
External	18–90	52.32 (13.72)
Expression	2–34	14.79 (5.88)
Social Support	6–30	18.61 (6.62)
Emotional Control	5–30	16.87 (5.38)

The Rasch Model reliability and separation indexes for items and persons supported the internal consistency of the FEEL-KJ item scores, with reliability scores about 0.7 and separation scores above 2. The reliability index (α, KR-20) for the items was 0.98 and the separation 6.27. As for the persons, the reliability index was 0.86 and the separation 2.47 [28,29].

Data’s fit to the Rasch Model, was assessed by calculating the infit MNSQ (unstandardized information-weighted mean square statistic), the outfit MNSQ (unstandardized outlier-sensitive mean square statistic), the point-measure correlations and the standard error estimates (See Table 5). Both infit and outfit MNSQ statistics provided evidence that supports the construct validity of the feel-kj questionnaire [34]. The infit and outfit mean values were close to the perfect fit value of 1 (1.01 and 1.02, respectively) [32]. Eight items (f13, f7, f25, f14, t7, t25, a25, and a21) were considered a misfit at the conservative range of 0.7 and 1.4 [30,31]. Items showed low standard error estimates ranging from 0.05 to 0.08 and reliable scores. Fifty-three items showed a good discrimination index with point-measurement correlations between the recommended values of 0.30 and 0.70 [32].

**Table 5:** Difficulty, infit MNSQ, outfit MNSQ, and point correlation statistics for the FEEL-KJ items.

Items	Items 1 to 45								Items 46 to 90				
	*N*	Item Difficulty	Model SE	Infit MNSQ	Outfit MNSQ	PMC	Items	*N*	Item Difficulty	Model SE	Infit MNSQ	Outfit MNSQ	PMC
f13	252	1.16	.08	1.36	1.54	.00	f8	254	−0.06	.05	1.02	1.02	.13
t13	251	1.08	.07	1.15	1.25	.10	f24	252	−0.07	.05	1.27	1.29	.15
f7	253	0.90	.07	1.30	1.43	−.04	t5	251	−0.08	.05	1.18	1.20	.13
a13	254	0.53	.06	1.26	1.33	.03	t10	251	−0.09	.05	0.84	0.84	.40
f9	253	0.47	.05	1.26	1.30	−.03	t24	251	−0.09	.05	1.22	1.24	.16
t23	251	0.46	.05	1.03	1.02	.31	a24	254	−0.10	.05	1.12	1.14	.19
f23	252	0.46	.05	1.07	1.08	.33	a27	252	−0.10	.05	1.08	1.09	.37
f25	250	0.45	.05	1.46	1.49	.02	f21	253	−0.11	.05	1.18	1.22	.11
a7	254	0.43	.05	1.29	1.33	.03	f1	254	−0.11	.05	0.89	0.89	.31
t22	251	0.42	.05	0.94	0.92	.40	t19	251	−0.12	.05	1.05	1.05	.43
f22	252	0.42	.05	1.18	1.19	.26	t2	251	−0.13	.05	1.06	1.06	.43
f14	252	0.42	.05	0.98	1.00	.26	a1	254	−0.14	.05	0.60	0.61	.45
t7	251	0.41	.05	1.55	1.58	−.03	a19	254	−0.15	.05	0.87	0.87	.49
t25	251	0.39	.05	1.47	1.54	−.10	a15	253	−0.16	.05	0.89	0.90	.33
a9	254	0.34	.05	1.10	1.14	.01	a20	253	−0.17	.05	0.82	0.85	.29
a29	253	0.34	.05	0.78	0.78	.30	a17	254	−0.17	.05	0.97	0.99	.44
t9	251	0.33	.05	1.20	1.22	.01	f26	252	−0.18	.05	1.34	1.35	.07
t14	250	0.33	.05	1.04	1.03	.36	f10	253	−0.18	.05	0.95	0.95	.46
a25	253	0.32	.05	1.43	1.45	−.02	f19	253	−0.18	.05	1.06	1.05	.42
a23	253	0.26	.05	1.00	1.00	.31	a3	254	−0.19	.05	0.96	0.98	.34
a30	253	0.26	.05	1.00	1.01	.16	t20	250	−0.20	.05	0.83	0.85	.38
f16	253	0.25	.05	0.94	0.95	.26	t15	251	−0.23	.05	0.96	0.97	.32
t30	250	0.24	.05	1.07	1.10	.10	f20	253	−0.26	.05	0.98	0.98	.34
a22	252	0.20	.05	1.20	1.21	.28	a26	254	−0.27	.05	1.34	1.36	.03
t29	250	0.20	.05	0.90	0.91	.31	t28	250	−0.27	.05	0.82	0.83	.43
a12	254	0.18	.05	0.75	0.75	.34	t26	250	−0.27	.05	1.03	1.02	.24
f30	252	0.15	.05	1.11	1.13	.23	f28	249	−0.27	.05	0.84	0.85	.39
t12	251	0.15	.05	0.82	0.83	.41	a28	252	−0.28	.05	0.74	0.75	.48
t21	251	0.13	.05	0.95	0.99	.24	t1	251	−0.33	.05	0.77	0.77	.45
a6	254	0.10	.05	0.75	0.76	.36	t27	250	−0.33	.05	1.03	1.04	.46
a10	254	0.09	.05	0.85	0.88	.36	f15	252	−0.35	.05	0.89	0.88	.52
a21	252	0.07	.05	0.91	0.94	.25	t11	251	−0.36	.05	0.67	0.67	.48
f29	250	0.06	.05	0.88	0.89	.26	t3	251	−0.36	.05	0.89	0.89	.55
a14	254	0.05	.05	1.10	1.11	.22	t17	250	−0.38	.05	1.04	1.04	.53
t8	251	0.05	.05	1.07	1.08	.18	a11	254	−0.38	.05	0.78	0.78	.37
f12	253	0.04	.05	0.89	0.89	.38	a18	254	−0.39	.05	0.77	0.77	.46
a5	254	−0.02	.05	1.19	1.22	.16	a4	254	−0.41	.05	0.99	1.00	.35
t16	251	−0.02	.05	0.86	0.88	.30	t4	251	−0.41	.05	0.96	0.95	.50
f2	254	−0.03	.05	0.98	0.98	.40	f11	254	−0.41	.05	0.82	0.80	.48
a8	254	−0.03	.05	0.82	0.83	.12	f27	251	−0.42	.05	1.00	0.99	.45
t6	251	−0.03	.05	0.86	0.86	.41	f3	253	−0.45	.05	0.84	0.83	.51
f6	251	−0.04	.05	0.72	0.72	.48	f4	253	−0.49	.05	0.95	0.93	.45
a16	254	−0.04	.05	0.91	0.93	.33	f17	253	−0.53	.05	1.02	0.99	.54
a2	254	−0.05	.05	0.97	0.97	.36	t18	250	−0.58	0.89	0.88	.48	
f5	254	−0.06	.05	1.33	1.36	.10	f18	253	−0.66	0.79	0.77	.48	
Model Mean for all items	252.3	0.00	.05	1.01	1.02								
Model SD for all items	1.50	0.34	.00	0.19	0.21								

Note*.* Items labelled as “a” correspond to anger items, as “t” to sadness, and as “f” to anxiety. Items are ordered by item difficulty level. Values considered as a misfit in relation to the 0.7 to 1.4 range are shown in boldface and underscored (Handley et al., 2008; Linacre, 2013).

**Fig. 1:**
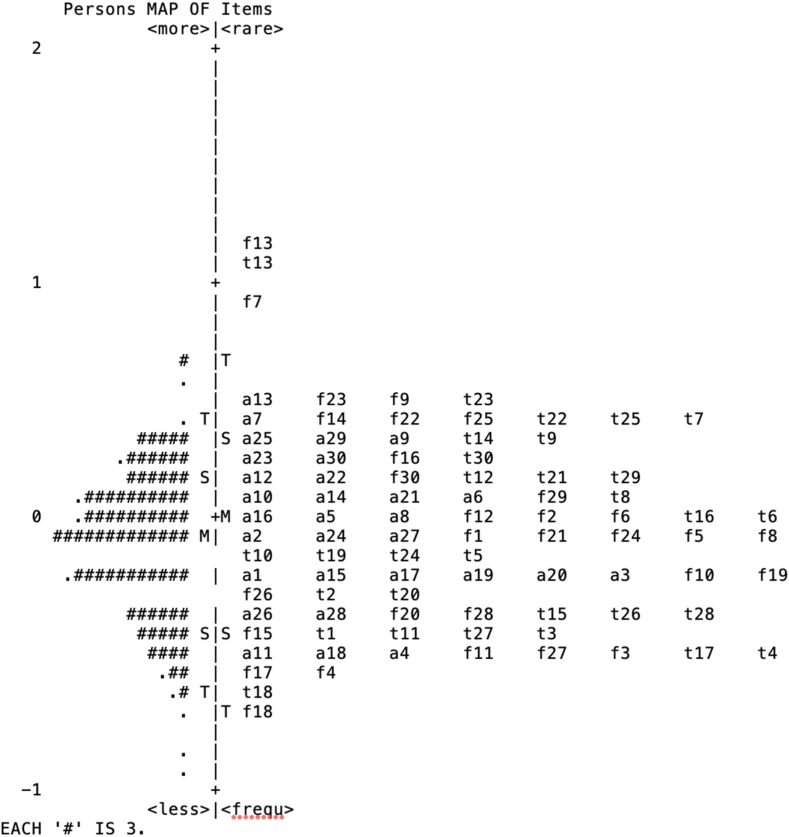
Person-item map for the FEEL-KJ in a Valencian Sample. Items Labelled as “a” Correspond to Anger Items, as “t” to Sadness, and as “f” to Anxiety. M = Mean, S = standard deviation T = 2 standard deviations.

The difficulty level of the items ranged from -.66 to 1.06 (Table 5). The person-item map depicts the participants' ability estimates (left) and the item difficulty estimates (right). The means of the person's ability estimates, and item difficulty estimates were close to 0 logits and both persons and items were symmetrically distributed across the map. The distribution of the persons and items in the map indicated that only the 2 bottom FEEL-KJ performers were not targeted by the questionnaire items. Extremely difficult items or extremely easy items do not discriminate among different levels of ability. Three items (i.e., f13, t13, and f7) out of 90 items of the FEEL-KJ questionnaire presented a difficulty level, >2 SD, that did not match the ability levels of the participants in the FEEL-KJ. No items were identified as too easy or as not matching the persons' ability levels (see Fig. 1).

**Fig. 2:**
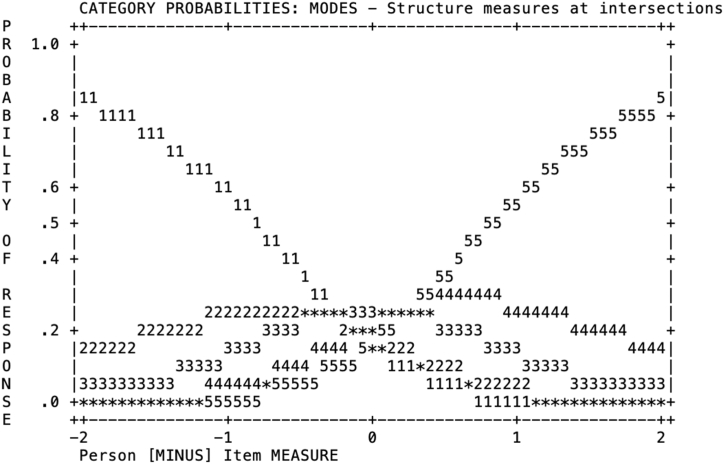
Category Probabilities for the FEEL-KJ five-point Rating Scale in our Sample.

The results of the partial credit model partially supported the well-functionality of the questionnaire response categories (see Table 6). The average scores (i.e., the probability of person's choosing a specific category related to their ability level represented by logits) were ordered, that meaning those persons with higher ability levels were more prompted to choose higher response categories, 4 or 5, and persons with lower ability levels were more likely to choose 1s or 2s. Infit and Outfit MNSQ statistics supported a good fit of the response categories with values below 2 and above −2 [35]. Nonetheless, the thresholds or step calibrations were close together and disorganized. Thresholds represents the point where it is equally likely to choose one of two adjacent response categories [33]. Fig. 2 shows the probability of a person choosing 2 or 3 is lower (-.37) than the probability of choosing 1 or 2 (-.13), and the probability of choosing either 3 or 4, or 4 or 5 were the same (0.25). These results might indicate that the terms used to translate the response categories are close in meaning (i.e., almost never, rarely, sometimes, often, and almost always) The proximity in meaning of the response categories might have made it difficult for the participants to understand the difference among response categories.

**Table 6:** Rating scale diagnostic statistics and parameters.

Category						
Label	Score	Observed Count (frequency)	Observed Average (logit)	Infit MNSQ	Outfit MNSQ	Structure Calibration
Almost never	1	4687	−0.33	1.02	1.02	None
Rarely	2	4147	−0.17	0.94	0.91	−.13
Sometimes	3	5444	−0.04	0.91	0.90	−.37
Often	4	4405	0.10	1.00	1.05	.25
Almost always	5	4020	0.22	1.02	1.07	.25

## 4 Discussion

From the results obtained, it can be argued that de FEEL-KJ scores are reliable. Infit and Outfit MNSQ suggest the construct validity of the scale, in line with [26]. When considering a conservative adjustment range (0.7–1.4) we observed that 8 items (f13, f7, f25, t7, t25, f14, a25, and a21) had a bad fit to the Rasch Model. If a more flexible range where to be adopted (0.5–1.5) only three items (f13, t7 and t25) would not have an appropriate fit. Point-measure correlations (PMC) values suggest that approximately 60 % of items had good discrimination values so they discriminated between different skill levels (emotional regulation). The people and items map suggest that virtually all items fit the different skill levels of the children and young people evaluated, only three items were above 2 SD.

Items that have a bad fit may owe these results to the translation presenting difficulties in moving the concept into Spanish.

The results state that, depending on the emotion, some strategies adapt to the Rasch model while others do not, which could imply that the same strategy is understood differently depending on the emotion to which it is applied (if number 21 adapts poorly to the model for anxiety and well for sadness it could mean that it is contemplated differently) this reinforces the notion of the need to study how each strategy adapts to every emotion. This difference could also be interpreted as a cultural product. One possible conclusion is that the results of lack of adaptation do not reflect only difficulties with translation, but that the underlying regulatory mechanism is totally different depending on what emotion is attempted to regulate. This may lead to the need to create specific mechanisms for each emotion (expressing emotion could be valid to regulate fear, but not to regulate anger) Most likely, the formulation of phrases is the problem and participants who answered the test did not recognize the descriptions of the regulatory strategies as the mechanisms they use to regulate themselves when considering a particular emotion.

Considering de increasing importance given to adaptative ER and the influence in psychopathological symptomatology that ER has in children [8] and adults [3] and in better social adjustment [7], these results added to previous validation of the scale [23] point in the direction that Feel-KJ will be a useful, valid and reliable tool to use in the speaking Spanish population, although some adjustment is necessary as far as translation is concerned. After addressing the translation difficulties mentioned before, this tool could have a great impact on the design of education intervention programs, providing information relevant to professionals who are helping children and adolescents with ER difficulties; addressing difficulties with the ER strategies related to specific emotions.

## Data availability statement

Access to datasets generated for this study will be granted upon request to the corresponding author.

## Ethics statement

This study is part of a project reviewed and approved by Catholic University of Valencia’s Ethics Committee of Investigation. Committee gave approval for this project on December the 20th^,^ 2017. Project’s code is UCV2017-2018-05. Legal guardian or next of kin written informed consent was necessary to take part in this study.

## CRediT authorship contribution statement

**Rodrigo M. Pazos Siri:** Conceptualization, Data curation, Formal analysis, Investigation, Writing – original draft, Writing – review & editing. **Catalina Morales Murillo:** Conceptualization, Formal analysis, Methodology. **María Dolores Grau Sevilla:** Conceptualization, Project administration, Validation, Writing – review & editing, Supervision. **Adoración Reyes Moliner:** Conceptualization, Supervision, Validation.

## Declaration of competing interest

The authors declare that they have no known competing financial interests or personal relationships that could have appeared to influence the work reported in this paper.
